# Clinical implications of respiratory ciliary dysfunction in heterotaxy patients with congenital heart disease: elevated risk of postoperative airway complications

**DOI:** 10.3389/fcvm.2023.1333277

**Published:** 2024-01-15

**Authors:** Tingting Zhao, Xianghui Huang, Weicheng Chen, Han Gao, Zhiyu Feng, Chaozhong Tan, Jingwei Sun, Xiaojing Ma, Weili Yan, Wei Sheng, Guoying Huang

**Affiliations:** ^1^ Children's Hospital Affiliated to Fudan University, Shanghai, China; ^2^ Shanghai Key Laboratory of Birth Defects, Shanghai, China; ^3^ Fujian Provincial Key Laboratory of Neonatal Diseases, Xiamen Children's Hospital Affiliated to Children's Hospital of Fudan University, Shanghai, Xiamen, China; ^4^ Bengbu First People's Hospital Affiliated to Bengbu Medical University, Hefei, Anhui, China; ^5^ Research Unit of Early Intervention of Genetically Related Childhood Cardiovascular Diseases, Chinese Academy of Medical Sciences, Shanghai, China

**Keywords:** postoperative airway complications, heterotaxy, congenital heart disease patients, respiratory ciliary dysfunction, primary ciliary dyskinesia

## Abstract

**Objective:**

Cardiac surgery in Congenital Heart Disease-Heterotaxy (CHD-HTX) patients often leads to increased postoperative airway complications. Abnormal respiratory ciliary function, resembling primary ciliary dyskinesia, has been observed. We expanded the sample size by retrospectively reviewing Ciliary Dysfunction (CD) in CHD-HTX patients to verify the increased risk of post-surgical respiratory complications.

**Methods:**

We conducted a retrospective review of 69 CHD-HTX patients undergoing cardiac surgery, assessing abnormal respiratory function using nasal nitric oxide (nNO) levels and nasal ciliary motion observed in video microscopy. Data collected included demographics, surgical details, postoperative complications, length of stay, ICU hours, salvage procedures, intubation duration, and mortality.

**Results:**

The CD and no-CD cohorts exhibited notable similarities in risk adjustment in Congenital Heart Surgery-1 (RACHS-1) risk categories, age at the time of surgery, and the duration of follow-up evaluations. We observed a trend toward an increased length of post-operative stay in the CD group (15.0 vs. 14.0; *P *= 0.0017). CHD-HTX patients with CD showed significantly higher rates of respiratory complications (70% vs. 44.4%; *P *= 0.008). There were no notable variances observed in postoperative hospitalization duration, mechanical ventilation period, or surgical mortality.

**Conclusion:**

Our findings suggest that CHD-HTX patients with CD may face an elevated risk of respiratory complications. These results offer guidance for perioperative management and serve as a reference for further pathological studies.

## Introduction

Heterotaxy is defined as any discordant arrangement of visceral organ due to the randomization of left-right axis during embryogenesis. Complex congenital heart disease is often associated with heterotaxy patients. Some studies have observed that approximately 3% of CHD patients present with heterotaxy (1) and 67% of heterotaxy patients have complex congenital heart disease (2). The connection between cardiovascular morphogenesis and heterotaxy relies on motile cilia for normal heart looping and left-right pattern determination. Motile cilia is also essential for patients with primary ciliary dyskinesia (PCD), which leads to recurrent respiratory symptoms due to abnormal airway ciliary motion (3). 50% PCD patients have situs inversus totalis (SIT), while 6.3%–12.1% exhibit heterotaxy (2, 4). Ciliary dysfunction (CD) is characterized by testing and observing nasal epithelial ciliary motion (CM) in video microscopy by scraping the nasal tissue.

Compliance with clinical findings and incidence, previous study has also shown that known genes, such as *Dnah5* and *Dnail, in* mutant mouse with primary ciliary disease, displayed a high incidence of situs ambiguous with complex congenital heart disease (CHD) (5, 6). Furthermore, in our recent publication, we demonstrated that WES results in heterotaxy patients revealed a high presence of the PCD-causing gene DNAH11 (7). This observation reflects the known requirement for the left and right pattern of the motor cilia function of the embryonic node, as well as the important role of cilia function in airway clearance in the respiratory epithelium.

To investigate the association between cilia dysfunction and CHD with heterotaxy, Nader Nakhleh et al. conducted a retrospective analysis of 43 HTX-CHD patients. Among them, 18 patients exhibited CD, as evidenced by abnormal ciliary motion and nasal nitric oxide (nNO) level below or borderline of PCD cutoff value, resulting in increased airway symptoms. Sequencing results also revealed that known PCD-causing genes were more frequently found in heterotaxy patients with CD compared to controls without CD (8). Additionally, CHD patients with heterotaxy experienced higher postoperative events, postsurgical mortality rates, and increased respiratory complications when compared to CHD patients without laterality defects with similar RACHS-1 complexity scores (9).

Building on the previous retrospective analysis, it was initially underestimated whether respiratory complications were correlated with ciliary dyskinesia. The question of whether the ciliary dysfunction in CHD patients with heterotaxy leads to worse postoperative events remains controversial. Brandon Harden et al. conducted a small sample study in a limited regional population, which was insufficient to explain and support the issue (10). In our study, we divided our CHD-HTX patients into CD or no-CD groups based on video microscopical observation. We tested the assumption that HTX-CHD patients with abnormal ciliary motion have increased postsurgical events and respiratory complications compared to HTX-CHD without CD in the Chinese population. Our study includes a total of 110 operative events, consisting of 36 CHD-HTX-CD patients in 56 surgeries and 33 CHD-HTX-no-CD in 54 surgeries. The study provides a theoretical basis for perioperative management.

## Materials and methods

### Patient population

This study was conducted with the approval of the Children's hospital of Fudan university Institutional Review Board. We retrospectively enrolled patients with heterotaxy with CHD who had undergone cardiac surgery at the Children's Hospital of Fudan University Cardiac Surgery Center between 2012 and 2020. We collected relevant information from electronic medical records (EMR). Detailed cardiac anatomical data were obtained from cardiac catheterization reports, echocardiographic reports, enhanced cardiographs, and CT scans, as well as operative reports. Thoracic and abdominal situs were determined based on chest radiography and abdominal ultrasonography. Preoperative evaluation included nasal nitric oxide measurements and video microscopy of respiratory epithelia ciliary cells. The medical staff involved in the study were blinded to ciliary function throughout the research.

### Definitions and inclusion criteria

In this study, heterotaxy was broadly defined as any thoracoabdominal organ discriminate from situs solitus. Our case-cohort consisted of patients with congenital heart defects undergoing cardiovascular surgery who also had laterality disorders. These laterality disorders included bronchial/pulmonary isomerism or inversus, midline or left-sided liver, midline or right-sided stomach, right-sided spleen, polysplenia or asplenia. Cardiovascular situs in CHD patients is classified as abnormal when they display dextrocardia/mesocardia, interrupted inferior vena cava, atrial isomerism/ambiguity/inversus, atrioventricular discordance, and superior/inferior ventricles (11). Cases with isolated CHD that didn't involve any of the cardiovascular situs anomalies mentioned above or other thoracoabdominal organ situs malformation were excluded.

### Nasal nitric oxide measurements

PCD assessment included measure of nasal nitric oxide (nNO) when abnormal nNO value has high sensitivity and specificity for PCD. We measured nasal NO (nNO) using a chemiluminescence nitric oxide analyzer (CLD88 SP, ECOPHYSICS AG) following established protocols (12, 13). Due to age-related variations in nNo levels, we divided participants into three age groups: <1 year old, 1- to 6-year-old, and >6 year old. For those over 6 years of age, we followed the velum-closure technique according to American Thoracic Society/European Respiratory Society guidelines. Normal nNO values are typically above 200 nl/min (12), whereas PCD patients exhibit values below 100 nl/min (14, 15). Values between 100 nl/min and 200 nl/min were considered borderline. For patients aged 1–6 years, nNO measurements were taken using tidal breath sampling (16). Healthy nNO value are generally >100 nl/min, while PCD patients often have values <50 nl/min (12). We categorized nNO levels as borderline low if they fell between 50 and 100 nl/min. In patients under 1 year old, specific nNO recommendations are limited due to measurement challenges. Phillip S. Adams et al. have developed a regression model to best estimate normative nNO values for infants less than 1 year old (17). We classified nNO values below the cutoff for each age group as abnormal, indicating CD.

### Nasal tissue sampling and ciliary motion analysis

In our study, we divided our patients into CD or non-CD groups based on nasal tissue and ciliary motion analysis. Nasal epithelial tissue samples were obtained by curettage of the inferior nasal turbinate before the operation, suspended in L-15 medium (Invitrogen, CA) for video microscopy was conducted using a Leica inverted microscope (Leica DMI300B) equipped with a 67× oil objective under differential interference contrast optics. Recordings were captured at a frame rate of 200 frames/s at room temperature, employing a 680 PROSILICA GE camera (Allied Vision, PA). Digital recordings were evaluated by an expert panel of co-investigators (Weicheng Chen and Tingting Zhao), ensuring they remained blinded to the subjects' phenotype, heterotaxy status, and nNO level. In order to avoid the effect of secondary ciliary dyskinesia, nasal tissue was cultured for re-assessment of ciliary motion after reciliation. The reciliated tissue was observed by video microscopy to assess whether CM was normal (CD) or abnormal (no-CD). We closely examined the ciliary beat pattern to assess potential abnormalities in ciliary motion. The process involved the use of slow-motion video playback to create tracings of the ciliary beat, with a minimum of 10 tracings analyzed for each subject. The classification of ciliary beat patterns encompassed normal, immotile, minimal residual movements, stiff, restricted, and circular (18). The quantification of ciliary beat frequency (CBF) was performed using ImageJ and Photoshop software.

### Data collection

Comprehensive demographic and preoperative data, encompassing visceral arrangement and medical history, were meticulously extracted from the medical records. Data regarding surgical procedures, including operation duration, Aortic Crossclamp Time, SVC Crossclamp Time, IVC Crossclamp Time, Cardiopulmonary Bypass, and Anesthetic duration, were documented. Additionally, we collected basic demographic information, such as age at the time of nNO and CM assessment, gender, race, gestational age, and diagnosis. RACHS-1 risk categories, known for their strong correlation with in-hospital mortality and postoperative hospital stay length, were used to facilitate relevant comparisons among different groups undergoing surgery for CHD/HTX.

Length of stay (LOS) parameters were also meticulously recorded, including total hospital stay duration, postoperative length of stay (PLOS), hours spent in the Cardiac Intensive Care Unit (CICU), and occurrences of in-hospital mortality. Postoperative mortality was characterized as any fatality within 30 days following the surgical procedure or during the patient's hospitalization after surgery. Measures related to postoperative respiratory outcomes encompassed the duration of mechanical ventilation in hours, the number of failed extubations, instances of prolonged ventilatory support, cases requiring salvage procedures, and the occurrence of fever. A patient's reintubation is characterized by failed extubation within 24 h. Viral infection was defined as positive nasal viral panel and clinical symptoms. Bacterial infection was defined as positive blood culture and clinical symptoms. The presence of fever combing with positive blood culture or nasal viral panel and clinical symptoms served as an indicator of postsurgical infection.

### Statistical analysis

We used summary statistics, including proportions and medians with interquartile ranges, to describe patient characteristics and postoperative outcomes. For comparisons, we employed *χ*^2^ tests, Fisher exact tests and Wilcoxon rank-sum tests for categorical and continuous variables. To address intrasubject correlations among surgical events, mixed-effects models were applied. For skewed continuous outcomes, we used linear mixed models on log-transformed data. Categorical outcomes were assessed with generalized linear mixed models. Multivariate analysis was conducted, controlling for baseline surgical characteristics. All tests were two-sided, and we set a significance level of *P* < 0.05. A more conservative threshold was used to adjust for multiple comparisons. SPSS was used for statistical analyses.

## Results

We enrolled 69 patients with HTX-CHD, which included 36 patients with heterotaxy with ciliary dysfunction (CD group) and 33 patients with heterotaxy congenital heart disease without ciliary function (no-CD group). Supplementary Tables E1, E2 present the structural heart position and laterality defects based on Van Praagh classification. Laterality abnormalities included variations in heart position (62%), stomach (61%), liver (77%), spleen (96%) and lung (100%). In the CD group, 22 patients (61%) had dextrocardia, while 12 (33%) had levocardia. Additionally, 7 patients (19%) had bilateral superior vena cava. Atrial and ventricular abnormalities included atrial septal defects (ASD) in 15 patients (42%) in the CD group and 15 patients (45%) in the no-CD group. Regarding conotruncal defects, there were various cases: 1 (3%) with Tetralogy of Fallot (TOF) in the CD group and 3 (9%) in the no-CD group, 1 (3%) with Pulmonary Atresia with Ventricular Septal Defect (PA/VSD) in the CD group and 3 (9%) in the no-CD group, 13 (36%) with D-transposition of the great arteries (D-TGA) in the CD group and 11 (33%) in the no-CD group, and 12 (33%) with Double Outlet Right Ventricle (DORV) in the CD group and 9 (27%) in the no-CD group (Table 1). For cases with a lack of or no record of imagological examination, findings showed 33% in stomach, 11% in liver, 27% in spleen, and 62% in lung. Specifically concerning the liver, in the CD group, 15 cases (42%) exhibited abnormalities compared to 14 cases (42%) in the no-CD group. The midline liver position was observed in 10 cases (28%) in the CD group and 8 cases (24%) in the no-CD group (Table 1).

**Table 1:** Cardiovascular anatomy and organ situs.

	CD, *n* (%) 36	No-CD, *n* (%) 33	Organ situs	CD, *n* (%) 36	No-CD, *n* (%) 33
Cardiac position			Stomach		
Dextrocardia	22 (61%)	19 (58%)	Normal	9 (25%)	9 (27%)
Mesocardia	2 (5.6%)	–	Opposite	14 (38%)	14 (42%)
Levocardia	12 (33%)	14 (42%)	No record	13 (36%)	10 (30%)
Venous anomalies			Liver		
Bilateral superior vena cava	7 (19%)	6 (18%)	Normal	8 (22%)	6 (18%)
Anomalous pulmonary venous return	3 (8.3%)	5 (15%)	Opposite	15 (42%)	14 (42%)
Atria and ventricles			Midline	10 (28%)	8 (24%)
Atrioventricular septal defect	9 (25%)	7 (21%)	No record	3 (8%)	5 (15%)
Atrial septal defect	15 (42%)	15 (45%)	Spleen		
Ventricular septal defect	17 (47%)	12 (36%)	Normal	2 (6%)	–
Atrioventricular valve atresia/stenosis	5 (14%)	5 (15%)	Right	16 (44%)	14 (42%)
Common atrium	2 (6%)	4 (12%)	Asplenia	7 (19%)	9 (27%)
Single ventricle morphology	13 (36%)	14 (42%)	Polysplenia	2 (6%)	–
Ventricular outflow and great vessels			No record	9 (25%)	10 (30%)
Double-out right ventricle	12 (33%)	9 (27%)	Lungs		
Pulmonary stenosis/atresia	22 (61%)	14 (42%)	Normal	–	–
Aortic stenosis/atresia	1 (3%)	4 (12%)	Inverted	10 (28%)	3 (9%)
Right aortic arch	10 (28%)	10 (30%)	Left isomerism	2 (6%)	-
Double-out left ventricle	1 (3%)	5 (15%)	Right isomerism	4 (11%)	7 (21%)
Conotruncal defect			No record	20 (56%)	22 (70%)
Tetralogy of Fallot, TOF	1 (3%)	3 (9%)	Van Praagh classification		
PA/VSD	1 (3%)	1 (3%)	{S,D,S}	1 (3%)	5 (15%)
D-TGA	13 (36%)	11 (33%)	{S,D,D}	–	4 (12%)
Double outlet right ventricle	12 (33%)	9 (27%)	{S,L,L}	5 (14%)	4 (12%)
L-TGA	4 (11%)	6 (18%)	{S,D,X}	2 (6%)	2 (6%)
			{S,D,L}	–	1 (3%)
			{I,L,L}	–	2 (6%)
			{I,D,D}	5 (14%)	1 (3%)
			{I,D,X}	2 (6%)	1 (3%)
			{A,D,D}	–	1 (3%)
			{A,D,S}	2 (6%)	2 (6%)
			{A,D,L}	1 (3%)	–
			{A,D,X}	2 (6%)	1 (3%)
			{A,L,D}	1 (3%)	–

### Baseline demographics stratified by ciliary motion and nNO Status

**Figure 1:**
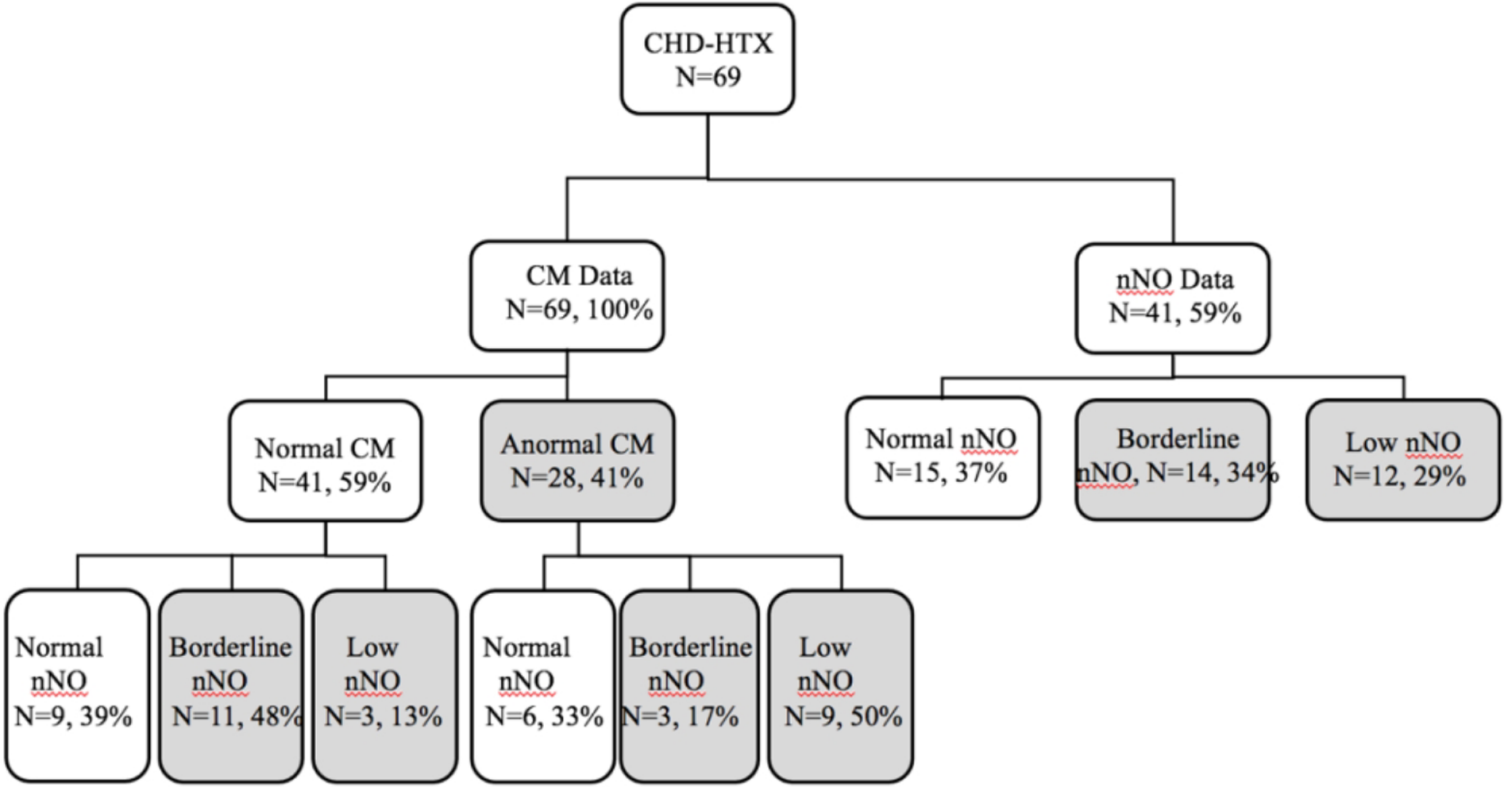
Enrollment data. Total congenital heart disease with HTX, *n* = 69. Results are presented as a flow chart. Shaded areas represent abnormal findings. CHD, congenital heart disease; nNO, nasal nitric oxide.

Nasal scrapes were obtained from all 69 participants for CM analysis, and nNO measurements were conducted in 41 (59%) participants. Abnormal CM was observed in 28 (41%) of the participants, with 14 (34%) showing borderline nNo values and 12 (29%) exhibiting low nNo values. Among the 41 who underwent both CM and nNO measurement, 12 showed abnormalities in both CM and low nNO (Figure 1). The nNO level in the patients with CD (86.1 nl/min) was significantly lower than in the patients without CD (119.16 nl/min).

### Clinical and surgical characteristics

Clinical and surgical outcomes are observed in Tables 2, 3. The patient age ranged from 6 days to 12 years at the time of surgery, with median age of 637.67 days for the CD group and 772.25 days for the no-CD group. Of the recruited patients, 65% were female, and 35% were male. All patients were of Asian descent and diagnosed at the Children's Hospital of Fudan university. The CD group underwent 56 cardiac surgical procedures, while the no-CD group underwent 54 surgeries. The median RACHS-1 scores for these operations, under both single and biventricular repair tracks, were 3.0 (range: 2.0–3.0) in the CD cohort and 3.0 (range: 2.0–3.0) in the no-CD cohort.

**Table 2:** Characteristics by surgical encounter.

	CD (*n* = 56)	No-CD (*n* = 54)	*P*-value
Age at surgery (d), median (IQR)	637.67 (286.00–1,487.50)	772.25 (240.25–1,491.00)	0.5601
Preoperative weight (kg), median (IQR)	9.8 (7.00–13.00)	11.43 (7.18–15.55)	0.2741
RACHS-1 risk category, median (IQR)	3.0 (2.0–3.0)	3.0 (2.0–3.0)	0.714
Length of hospitalization (d), median (IQR)	28.0 (19.5–34.7)	24.0 (20.0–33.5)	0.1141
Length of post-operative stay (d), median (IQR)	15.0 (10.0–21.0)	14.0 (11.0–18.8)	0.0017
Hours in ICU (h), median (IQR)	83.5 (44.5–170.5)	96 (48.0–164.25)	0.6803
No. of Salvage, *n* (%)	2 (3.6%)	5 (9.1%)	0.266*

*P*-values obtained by Wilcoxon rank-sum test for continuous variables or Pearson c2 test for categorical variables. nNO, nasal nitric oxide.

* *P*-value determined by the Fisher exact test.

**Table 3:** Comparison of RACHS-1 scores in CD and Non-CD.

RACHS-1 category	CD *N*	%	No-CD *N*	%	*P-*value
1	1	1.8%	0	0.0	0.714
2	24	42.9%	27	50.0%	
3	31	55.4%	26	48.1%	
4	0	0.0	1	1.9%	
5	0	0.0	0	0.0	
6	0	0.0	0	0.0	
Total	56		54		

The Wilcoxon rank-sum test was used to compare the distribution of the RACHS-1.

### Comparison of surgical and postsurgical outcomes

Table 4 demonstrates that there were no statistically significant differences in age at the time of surgery, preoperative weight, or sex between the CD group and the no-CD group. However, there was a trend toward younger age at surgery in CD patients than no-CD patients, with median age of 637.67 vs. 772.25 respectively. The median length of hospitalization for the CD group was 28 days (IQR, 19.5–34.7 days), which was not statistically distinguished from the 54 surgical encounters of the no-CD group, while the no-CD group with median of 24 days (IQR, 20.0–33.5 days), which was not significantly different. The median length of post-operative stays in the CD cohort (15.0 days, IQR, 10.0–21.0) was longer than in the no-CD cohort (14.0 days, IQR, 11.0–18.8), and this difference was statistically significant (*P* = 0.0017). However, there were no statistically significant differences in the hours spent in the ICU between the CD cohort (median, 83.5 h) and the no-CD cohort (median, 96 h). The number of postsurgical salvage cases in the CD group was 2 patients (3.6%) less than that of no-CD group (*n* = 5, 1%), with no statistically significant difference seen (*P* = 0.438) (Table 3). There was no difference in the length of surgery, Aortic Crossclamp time, SVC Crossclamp time, IVC Crossclamp time, Cardiopulmonary Bypass, or Anesthetic Duration between patients stratified by the CD group or no-CD group (Table 4).

**Table 4:** Comparison of surgery in CD and Non-CD.

	CD (*n* = 56)	No-CD (*n* = 54)	*P-*value
Length of surgery, median (IQR)	175.0 (150.00–220)	180.0 (144.15–207.63)	0.4032
Aortic Crossclamp Time, median (IQR)	8.6 (0.0–33.0)	15.0 (0.0–41.75)	0.6921
SVC Crossclamp Time, median (IQR)	28.0 (8.3–49.0)	41.75 (18.37–55.0)	0.1918
IVC Crossclamp Time, median (IQR)	0.0 (0.0–40.0)	27.25 (0.0–48.0)	0.0969
Cardiopulmonary bypass, median (IQR)	86.0 (67.0–115.0)	78.75 (53.5–100.5)	0.2234
Anesthetic duration, median (IQR)	205 (180.0–278.0)	210.0 (178.75–243.38)	0.1738

Data are median (interquartile range) or *n* (%). *P*-values obtained from Mann–Whitney and *χ*^2^ for continuous and categorical variables.

### Comparison of morbidity and mortality

A follow-up analysis was conducted on postsurgical patients who survived their last surgery. Death within 30 days post-hospitalization was considered a case. Two death cases were observed in the CD cohort, and six cases in the no-CD group. Notably, parents of patient #5,119, #5,876, and #51,372 in the no-CD group proactively discontinued treatment (Supplementary Table E2). Statistical analysis showed no statistical difference in postoperative mortality (≤30 days) between the CD group and no-CD group (Table 5).

**Table 5:** Comparison of morbidity and mortality between CD and No-CD.

	CD (*n* = 36)	No-CD (*n* = 33)	*P*-value
Death ≤30days, *n* (%)	2 (5.6%)	6 (18.2%)	0.14
Neonatal respiratory distress, *n* (%)	0 (0)	0 (0)	–

*P*-value determined by the Fisher exact test.

### Comparison of respiratory outcomes

In the case of the 56 CHD surgical procedures, the median duration of postoperative mechanical ventilation was 22.5 h (IQR, 10.25–93). Among these surgical encounter, one patient required a prolonged ventilator course (≥10 days) (Table 5). In contrast, the no-CD group, with 54 surgical outcomes, had a median length of intubation of 21.5 h (IQR, 10.82–69.25), with no significant difference in the duration of mechanical ventilation between the CD and no-CD group (*P *= 0.37). Only The patient in the CD cohort required reintubation. The number of postoperative fever (≥38.5°C) showed no statistical difference between the two groups, with the 18 instances of postoperative fever in the 56 surgical encounters compared to 12 instances in the no-CD group (Table 6).

**Table 6:** Respiratory outcome measures by surgical encounter.

	CD (*n* = 56)	No-CD (*n* = 54)	*P*-value
Length of mechanical ventilation (hr), median (IQR)	22.5 (10.25–93)	21.5 (10.82–69.25)	0.3700
Prolonged ventilator course (≥10days), *n* (%)	1 (2.7%)	0 (0)	>0.99
No. failed extubations, *n* (%)	1 (1.8%)	0 (0)	>0.99
Fever (≥38.5°C), *n* (%)	18 (32.1%)	12 (22.2%)	0.243
Respiratory complication, *n* (%)	39 (70%)	24 (44.4%)	0.008

Data are median (interquartile range) or *n* (%). *P*-values obtained from Mann Whitney and *χ*^2^ for continuous and categorical variables.

The frequency of respiratory complications was compared between the 2 groups. Respiratory complications were identified through postoperative chest x-rays, encompassing pleural effusion, atelectasis, pneumothorax, pleural exudation, pneumonia, and delayed sternal closure. Notably, respiratory complications occurred 39 (70%) of the 56 cardiac surgeries in the CD cohort, whereas the no-CD group experienced these complications in only 24 (44.4%) of their surgeries, signifying a significantly lower frequency in the latter group (*P *= 0.008) (Table 6). In-depth subgroup analysis of respiratory complications, excluding those not solely attributed to cilia dysfunction (such as pleural effusions and pulmonary edema), consistently demonstrated a significantly higher prevalence of respiratory problems within the congenital heart disease (CD) cohort (*P < *0.02).

## Discussion

The mucociliary clearance system serves as a pivotal physiological mechanism responsible for the removal of inhaled foreign substances and endogenously generated secretions from the respiratory airways. Ciliary dysfunction, a critical component of this system, manifests in two primary forms: PCD, which is a congenital disorder characterized by impaired mucociliary transport, and secondary ciliary dyskinesia, which may develop subsequent to injuries such as respiratory infections. It is noteworthy that secondary ciliary dyskinesia presents distinct ultrastructural abnormalities in contrast to the primary form of the disorder, PCD.

In our research investigation, patients were categorized into groups based on the presence or absence of ciliary dysfunction, which encompassed primary ciliary dyskinesia and secondary ciliary dysfunction. Ciliary motion can be induced by secondary ciliary dyskinesia, such as from respiratory infection and allergy to environmental insults, but in our study, this can be eliminated by observing the ciliary motion after culturing and reciliation of nasal tissue (19–21). Although some individuals, particularly those with heterotaxy and CHD, may not exhibit overt structural deficiencies in their cilia ultrastructure, the observation of abnormal ciliary motion through microscopic analysis suggests that their ciliary response to environmental stimuli is fragile. Significantly, our findings indicate that this ciliary dysfunction is potentially reversible when the affected tissue is removed from the infected or inflamed environment. Neonatal respiratory distress and bronchiectasis, as well as respiratory symptom, are frequently observed in individuals with PCD. Early research has provided insights indicating that an escalation in sinopulmonary symptoms does not appear to be contingent on the presence of heterotaxy. This implies that patients with CHD face an elevated risk for respiratory ailments linked to reduced nNO levels or abnormal ciliary motion, rather than the condition of heterotaxy itself (8). In an endeavor to gain a more comprehensive understanding of whether abnormal ciliary motion is linked to CHD-HTX, we conducted an observational study. Our findings revealed that 28 out of 69 (41%) of CHD-HTX patients presented CD. The ciliary motion anomalies identified in these CHD-HTX patients closely resembled those classically seen in PCD, characterized by the presence of stiff, dyskinetic or wavy ciliary beat. Remarkably, among patients with CD, approximately 50% exhibited low nNO levels, while 17% exhibited nNO values in proximity to the PCD diagnostic threshold. Notably, the mean nNO values of the CHD-HTX patients with CD exceeded those of their counterparts without CD. This collection of observations strongly suggests that individuals with CHD and heterotaxy may harbor CD, which exhibits characteristics overlapping with PCD, including both abnormal ciliary motion and reduced nNO levels.

In a previous study, researchers analyzed the postoperative outcomes of 13 patients with heterotaxy and CHD who displayed CD, comparing them to 14 patients with heterotaxy and CHD but without CD. This earlier investigation revealed an increased risk of respiratory complications in those with CD (10). Nevertheless, it is essential to acknowledge that the limited sample size of this study could have introduced potential errors and inaccuracies. To rectify this limitation, we expanded the sample size and conducted a prospective assessment involving 36 heterotaxy patients with CHD and confirmed CD who underwent cardiac surgery. These individuals were juxtaposed with a control group comprising 33 CHD-heterotaxy patients without CD. Importantly, it is noteworthy that both the CD and no-CD groups were equivalent in terms of their Risk Adjustment for Congenital Heart Surgery (RACHS-1) scores, signifying that they underwent cardiac surgeries of comparable complexity. In contrast, the CD group exhibited increased incidences of respiratory complications following surgery and required extended post-operative hospital stays when compared to the no-CD group. This observation implies that patients with heterotaxy and CHD may be prone to experiencing respiratory complications due to underlying ciliary dysfunction. This hypothesis finds support in the notable occurrence of heterotaxy in a murine model of PCD, as documented in a previous study (5). Furthermore, our understanding is bolstered by research that has validated the significant roles played by DNAH11, DNAH5, and DNAI genes in encoding key components of respiratory and nodal ciliary outer dynein arm proteins (22, 23). These genetic factors have been established as contributors to the development of heterotaxy syndrome and CHD.

In light of our meticulous clinical observations and comprehensive research findings, our study contributes significantly to the nuanced understanding of the intricate associations between PCD, heterotaxy, and CHD. Postoperative evaluation of 36 CHD-HTX with CD undergoing cardiac surgery showed that high risks related to respiratory deficiency comparing to 33 CHD-HTX without CD. CHD-HTX with CD showed a greater need for mechanical ventilation, prolonged ventilator course and more failed extubation. We noted that no difference in number of death and neonatal respiratory distress between 2 groups. By raising awareness among healthcare practitioners regarding the potential risk of ciliary CD in patients with CHD, we aim to expedite the diagnostic process, ultimately leading to enhanced healthcare outcomes. Our investigation unearthed a noteworthy increase in the incidence of postoperative respiratory complications in the CD group, which was substantiated by a thorough analysis of chest x-rays conducted during the hospitalization period. These complications encompassed a range of conditions, including pleural effusion, atelectasis, pneumothorax, pleural exudation, pneumonia, and delayed sternal closure. It is essential to emphasize that ciliary dyskinesia, when considered in isolation, is not directly causative of conditions such as pleural effusion, stridor, and pulmonary edema. However, CD solely cannot account for pleural effusions, most likely to occur respiratory complication in the study to be further studied.

In our study, there was no difference in the length of surgery, Aortic Crossclamp time, SVC Crossclamp time, IVC Crossclamp time, Cardiopulmonary Bypass, or Anesthetic Durhuaation between patients stratified by the CD group or no-CD group, which keep consistency with RACHS-1 between CD and no-CD groups in case of surgery influence. With comparison to no-CD group, there were trends that CD groups took more time in the length of hospitalization, hours spent in the ICU, or the occurrence of prolonged ventilatory courses, though not statistically significant. In the case of the 56 CHD surgical procedures, the median duration of postoperative mechanical ventilation was 22.5 h more than 21.5 h in the no-CD group. Among these surgical encounter, one patient required a prolonged ventilator course (≥10 days) in CD group. Only the patient in the CD cohort required reintubation. The number of postoperative fevers showed 18 instances in the 56 surgical encounters compared to 12 instances in the no-CD group, though no significant difference, which implied that HTX with CD were more susceptible to external factor. Given single center of the present study, further analysis of clinical treatment of patients is needed to further assess the impact of CD on these outcome parameters.

To provide a more precise analysis, a subgroup assessment was meticulously conducted, systematically excluding complications that could not be solely attributed to ciliary dysfunction (specifically, pleural effusions and pulmonary edema). Remarkably, this rigorous subgroup analysis demonstrated significantly higher rates of adverse respiratory outcomes within the CD cohort (*P* < 0.02). The observed increase in the median duration of postoperative hospital stays for the CD cohort aligns with our expectations, reflecting the extended period required for the recovery and recuperation of these patients. This underscores the intricate interplay between ciliary dysfunction, heterotaxy, and CHD, and the profound impact it has on clinical outcomes.

### Limitation

In our study, no statistically significant increases were observed in the length of hospitalization, hours spent in the ICU, duration of postoperative mechanical ventilation, or the occurrence of prolonged ventilatory courses. These results diverge from earlier research that reported a rising trend in the duration of postoperative mechanical ventilation and prolonged ventilatory courses (10). This inconsistency might be linked to differences in the racial backgrounds and sample sizes of the populations studied.

## Conclusion

In summary, our study not only advances our understanding of the intricate interplay between PCD, heterotaxy, and CHD but also underscores the need for preoperative screening of ciliary dysfunction in heterotaxy patients with CHD. This proactive approach can significantly enhance postsurgical respiratory therapy and ultimately improve the overall well-being of these patients (6).

## Data Availability

The original contributions presented in the study are included in the article/**Supplementary Material**, further inquiries can be directed to the corresponding authors.
