# Supplementary material for: Clinical implications of respiratory ciliary dysfunction in heterotaxy patients with congenital heart disease: elevated risk of postoperative airway complications
Source: Front Cardiovasc Med. 2024 Jan 15;10:1333277. doi: 10.3389/fcvm.2023.1333277 (PMC10825948; doi:10.3389/fcvm.2023.1333277)
Supplement: Supplementary file 1 [file Datasheet1.pdf]

Supplement

TABLE E1. Heterotaxy patients with CHD and airway ciliary dysfunction

Patient no.	Age (d)	Complete cardiovascular anatomy	Procedure (RACHS-1)	Other laterality defects	nNO (nL/mi)
5020	147	{I, L, I} Dextrocardia, VSD,DA	VSD repair (2)	stomach/liver/spleen inversus	46.65
5026	370	{I, L, I} Dextrocardia, FSV,TAPVD, DORV	TAPVD>30d repair, BT shunt, Transection of pulmonary artery, (3)	Liver/spleen inversus	30.6
5027	658	{A, D, I} Right atrial isomerism, BLSVC,SV,DORV,PVS	Bilateral bidirectional Glenn (2)	-	13.98
5028	580	{I, L, I} Dextrocardia, atrial situs inversus, TGA,VSD,DORV,BLSVC,DA	Bilateral bidirectional Glenn, pulmonary artery banding (3)	Bronchial inversus, stomach/liver/spleen inversus	53.85
5031	276<br>1250	{I, L, A} Dextrocardia, CAVC,DORV,ASD,VSD,PS	Bilateral bidirectional Glenn (2)<br>Fontan (3)	-	37.8
5032	4463	{I, D, D}SV, SA, CAVSD,	Fontan (3)	Bronchial inversus,	225.3

		TGA, PS		Middle liver, stomach inversus	
5033	1884	{S, D, A} Dextrocardia, SV, PA, ASD	Fontan (3)	-	226.2
5038	1642<br>1913	{I,D,D}Levocardia, atrial situs inversus, SV,TA,PA,TGA,ASD,DA	Glenn (2)<br>Fontan, RV-PA conduit (3)	Bronchial inversus , stomach/liver/spleen inversus	177.6
5041	254<br>596<br>1891	{S,L,L}Dextrocardia, ventricular situs inversus, PA,TGA,VSD, pulmonary artery dysplasia	BT shunt (3)<br>Glenn (2)<br>Fontan, RV-PA conduit (3)	-	177.83
5042	3330	{S,L,D}Dextrocardia, ventricular situs inversus, MA,VSD,ASD,HLHS,DORV, D-TGA,PS	Glenn (2)	Middle liver	0
5043	418<br>1559	{A,L,S}Dextrocardia, ventricular situs inversus, PA,CAVC,ASD	Glenn (2)<br>Fontan (3)	Bronchial right isomerism, middle liver, asplenia	21.75
5044	658	{I,L,I}Dextrocardia, CAVC,DORV,VSD,ASD	bidirectional Glenn (2)	Bronchial inversus , stomach/liver/spleen inversus	240.38
5048	348	{S,D,A}Dextrocardia, SV,PAVC,DOLV,PS	bidirectional Glenn (2)	-	45.15
5053	81<br>2894	{I,L,I}Dextrocardia,D-TGA,AVSD,PS	bidirectional Glenn (2)<br>Fontan (3)	Middle liver, asplenia	133.05
5063	148	{A,D,L}Dextrocardia,	bidirectional Glenn,	Middle liver, asplenia	14.475

		SV,TGA,CAVC,DA	pulmonary artery banding (3)		
5066	1393	{I,D,D}Dextrocardia, atrial situs inversus, DORV, ASD,PS	bidirectional Glenn, Transection of pulmonary artery (2)	Liver/spleen inversus	121.05
	1683		fontan (3)		
5071	56	{S,D,S}ASD,PAPVD	PAPVD surgery, ASD surgery (1)	Left right isomerism, Middle liver, spleen inversus, polysplenia	54
5087	937	{I,D,A}Dextrocardia, DA,DORV,TGA,CAVC,TAPVD,PS	bidirectional Glenn, repair of TAPVD >30d (2)	Middle liver, asplenia	174.075
5126	286	{S,L,L}L-TGA,PA,VSD,ASD	Glenn (2)	-	0
5127	198	{I,L,I}Dextrocardia, DORV,BLSVC	Repair of double-outlet right ventricle (3)	stomach/liver/spleen inversus	0
5129	1324	{I,D,D}Levocardia, SV,MA,DORV,PS	Glenn (2)	Bronchial inversus, stomach/liver/spleen inversus	0
	2045		Fontan, RV-PA conduit (3)		
5130	414	{A,D,A}Right atrial isomerism, SV,CAVC,TGA,PS	Glenn (2)	Bronchial right isomerism, asplenia	0
	1321		Mitral valvulopasty, tricuspid valvuloplasty (3)		
5138	142	{S,L,S}Mesocardia, ventricular situs inversus, TGA,PA,VSD,BLSVC,DA	BT shunt (3)	-	0
5145	1369	{A,D,S}Dextrocardia, AVSD, Right atrial isomerism, PS,DA	Glenn (2)	Middle liver, asplenia	0
	1703		Atrioventricular valve annuloplasty (3)		
	2086		Atrioventricular valve replacement		

			(3)		
5204	90<br>197<br>1243	{I,L,S}Levocardia, SV, ventricular situs inversus, MA,TGA/PS,ASD,DA	BT shunt (3)<br>Glenn (2)<br>Fontan (3)	Bronchial inversus, stomach/spleen inversus, middle liver	6.2175
5635	331<br>1012	{S,L,L}L-TGA, Ebstein/TR	pulmonary artery banding (3)<br>mitral valve replacement (3)	-	48.6
5707	6<br>188	{I,D,S}Levocardia, SV,PA,CAVC,ASD	BT shunt (3)<br>Glenn, mitral valvuloplasty (3)	Liver/spleen inversus, bronchial inversus	13.5
5831	125<br>347<br>1441	{S,L,L}L-TGA,VSD	pulmonary artery banding (3)<br>Glenn (2)<br>Fontan (3)	-	0
51314	469	{S,L,L}L-TGA, VSD, ASD, PS, right superior vena cava to the left atrium	Glenn (2)	bronchial inversus, stomach/liver/spleen inversus	0
51454	267<br>478	{A,L,D}LAI, ventricular situs inversus, DORV,VSD,TGA,PS,ASD,DA ,BLSVC	BT shunt (3)<br>Glenn (2)	Stomach/spleen inversus, middle liver	1.42
51485	314	{A,L,I}Dextrocardia, Right atrial isomerism ,SV,CAVC,TGA,PA ,ASD	Glenn (2)	Brochial right isomerism, middle liver, asplenia	0
51513	120<br>1238	{A,D,S}SV,MA,SA,VSD	Glenn (2)<br>Fontan (3)	Bronchial inversus, stomach/liver/spleen inversus	0
51518	65	{I,D,D}Dextrocardia,	pulmonary artery banding (3)	Bronchial inversus,	23.7

	375	DORV,DA,VSD, bicuspid aortic valve	repair of DORV (3)	stomach/liver/spleen inversus	
51531	1647	{I,L,I}Dextrocardia, TOF, BLSVC, situs inversus	Repair of TOF (2)	Brochial right isomerism, stomach/liver/spleen inversus	79.13
51569	101	{I,L,I}Dextrocardia, situs inversus, BLSVC	repair of DORV (3)	Left right isomerism, stomach/liver/spleen inversus ,polysplenia	1.53
51834	780	{I,L,I}Dextrocardia, PA/VSD,ASD	Glenn (2)	liver/spleen inversus	184.8

Heterotaxy patients with CHD and airway ciliary dysfunction

All patients had abnormal cilia motion by video microscopy. ASD, Atrial septal defect; A-V, atrioventricular; AVC, atrioventricular canal defect; BT, Blalock Taussig; DORV, double-outlet right ventricle; HLHS, hypoplastic left heart syndrome; HLV, hypoplastic left ventricle; IVC, inferior vena cava; LA, left atrium; LIVC, left inferior vena cava; LPA, left pulmonary artery; LSVC, left superior vena cava; MAPCA, multiple aortopulmonary collateral; nNO, nasal nitric oxide; PA, pulmonary atresia; PAPVR, partial anomalous pulmonary venous return; PDA, patent ductus arteriosus; PS, pulmonic stenosis; RA, right atrium; RACHS-1, Risk Adjustment in Congenital Heart Surgery-1; RSVC, right superior vena cava; RV, right ventricle; SVC, superior vena cava; TAPVR, total anomalous pulmonary venous return; TOF, tetralogy of Fallot; VSD, ventricular septal defect. *Van Praagh classification (1977).

TABLE E2. Heterotaxy patients with CHD without airway ciliary dysfunction

Patient no.	Age（d）	Complete cardiovascular anatomy	Procedure (RACHS-1)	Other laterality defects	nNO (nL/mi)
5030	4695	{S, D, S} Dextrocardia, VSD, ASD	Arial septal defect and ventricular septal defect repair (2)	stomach/Liver/spleen/pancreas inversus	122.1
5037	602	{I, L, I} Dextrocardia, VSD, DA, BLSVC	VSD repair (2)	stomach/Liver/spleen inversus	122.475

5046	2376<br>2742	{I, L, L} Dextrocardia, atrial inversus, L-TGA,VSD,ASD,DORV	Glenn (2)<br>Fontan, RV-PA conduit (3)	Liver/spleen inversus	180.675
5049	196	{S, D, L} Dextrocardia, CAVC, TGA,SV,PS,DA	Glenn (2)		55.725
5056	286<br>3226	{I, D, D} Dextrocardia, atrial inversus, DOLV/TGA,SV,ASD,PVS	Glenn (2)<br>Fontan (3)	Stomach inversus, middle liver, asplenia	197.55
5072	253	{S, L, D} ventricular situs inversus, DORV, CAVC,TAPVD,ASD,DA	Glenn (2)	Middle liver, asplenia	43.65
5075	1829<br>2185	{A, D, A} Right atrial isomerism, SV,CAVC,DORV,TGA,PVS,DA	Glenn (2)<br>Fontan (3)	Middle liver, asplenia	0
5078	203<br>326<br>1413	{S, D, D} Dextrocardia, criss-cross heart, DORV,VSD,DA	PA banding (3)<br>Glenn (2)<br>Fontan (3)	Liver/spleen inversus	286.8
5088	728<br>1203<br>2000	{S, D, A} Dextrocardia, SV, MA,HLHS,DORV,TGA	BT shunt (3)<br>Glenn (2)<br>Fontan (3)		120.825
5101	40<br>547	{I, L, A} Dextrocardia, SV, MA,HLHS,SA,DORV/TGA,TAPVD	Repair of TAPVD >30d, PA banding (3)<br>Glenn (2)	Bronchial right isomerism, stomach/liver/spleen inversus	46.575
5102	1437	{I, L, I} Dextrocardia, CAVC, PA, ASD	Glenn (2)	stomach/Liver/spleen inversus	0
5103	211<br>1315	{I, D, I} Dextrocardia, SV, TGA,PS	Glenn (2)<br>Fontan (3)	Liver/spleen inversus	0
5115	3017	{A, D, D} Dextrocardia, right atrial	Glenn (2)	Bronchial right	0

	3268	isomerism, SV,CAVC,SA,DOLV/TGA,PS	Annuloplasty (3)	isomerism, stomach inversus, middle liver, asplenia	
5119	495<br>1777	{A, L, S} Right atrial isomerism, ventricular situs inversus, SV, MA,SA,VSD,DOLV,PS,TR	Mitral valvuloplasty, Glenn, PA banding (3)<br>Fontan (3)	Bronchial right isomerism, middle liver, aspenia	0
5155	87<br>259	{S, D, S} Levocardia, SV, MA, PS, cor triatriatum, BLSVC	BT shunt (3)<br>Glenn (2)	Bronchial right isomerism, stomach inversus, middle liver, aspenia	0
5163	308<br>689<br>1381	{S, L, L} L-TGA, PVS, PS, VSD, ASD, DA	BT shunt (3)<br>Glenn (2)<br>Fontan (3)		0
5188	102<br>246	{A, D, S} Right atrial isomerism, CAVC, SV, DOLV,ASD,PS,TAPVD,	Repair of TAPVD >30d (2)<br>Glenn (2)	Bronchial right isomerism, stomach inversus, middle liver, asplenia	0
5191	1129<br>1484	{S, L, D} ventricular situs inversus, SV, DORV,TGA,ASD	Glenn (2)<br>Fontan (3)		83.175
5238	82	{I, L, I} Dextrocardia, VSD, ASD	VSD/ASD repair (2)	Stomach/liver/spleen inversus	0
5309	2640<br>3673	{S, L, L} L-TGA	PA banding (3)<br>Doule switch (4)		0
5876	189	{S, D, D} TOF, criss-cross heart, DA	Total repair of TOF (2)		0
5945	762<br>1121	{I, L, S} Levocardia, atrial inversus, PA/VSD,ASD,DA	BT shunt (3)<br>RV-PA conduit replacement	Bronchial inversus, stomach/Liver/spleen	0

			(3)	inversus	
51039	205	{S, D, S} TOF, dextrocardia	Total repair of TOF (2)		95.1
51211	781	{I, L, I} Dextrocardia, SV/ventricular torsion, DOLV, PS, ASD, BLSVC	Glenn (2)	Bronchial inversus, stomach/liver/spleen inversus	83.25
51241	252	{S, L, L} L-TGA, ASD, VSD, PS	BT shunt (3)	stomach/liver/spleen inversus	0
51365	507<br>1020	{I, L, L} L-TGA, VSD, ASD, PS, DA, situs inversus	Glenn (2)<br>Fontan, RV-PA conduit (3)	Bronchial inversus, stomach/liver/spleen inversus	0
51372	53	{S, D, D} CAVC, SV, D-TGA, APVD, PA	Total repair of TAPVD>30d, BT shunt (3)	Middle liver, asplenia	0
51468	553	{S, D, S} Dextrocardia, VSD	VSD repair (2)		0
51486	181<br>1418	{S, D, D} Dextrocardia, DORV, ASD, BLSVC	Glenn (2)<br>Fontan, RV-PA conduit (3)		0
51557	303	{S, D, S} Dextrocardia, TOF	Total repair of TOF (2)	Liver/spleen inversus	26.175
51766	1653	{S, L, L} Dextrocardia, ventricular inversus, L-TGA, VSD,PVS,BLSVC	PA banding (3)	Bronchial right isomerism	76.05
51791	1962	{I, L, A} Dextrocardia, DORV, TGA, BLSVC, DA, PS	Fontan (3)	stomach/liver/spleen inversus	186.5
51793	554<br>1339	{A, D, S} Right atrial isomerism, SV, MA, HLHS, TGA, SA, TAPVD, ASD	Glenn (2)<br>Fontan, RV-PA conduit (3)	Bronchial right isomerism, asplenia	180

Heterotaxy patients with CHD without airway ciliary dysfunction

All patients had normal cilia motion by video microscopy. AS, Aortic stenosis; ASD, Atrial septal defect; AV, atrioventricular; AVC, atrioventricular canal defect; BT, Blalock Taussig; CS, coronary sinus; DILV, double-inlet left ventricle; DORV, double-outlet right ventricle; HLHS, hypoplastic left heart syndrome; HLV, hypoplastic left ventricle; HRV, hypoplastic right ventricle; IVC, inferior vena cava; LA, left atrium; LIVC, left inferior vena cava; LSVC, left superior vena cava; MAPCA,

multiple aortopulmonary collateral; MS, mitral valve stenosis; nNO, nasal nitric oxide; PA, pulmonary atresia; PAPVR, partial anomalous pulmonary venous return; PDA, patent ductus arteriosus; PS, pulmonic stenosis; RA, right atrium; RACHS-1, Risk Adjustment in Congenital Heart Surgery-1; RIVC, right inferior vena cava; RSVC, right superior vena cava; RV, right ventricle; SVC, superior vena cava; TAPVR, total anomalous pulmonary venous return; TGA, transposition of the great vessels; TOF, tetralogy of Fallot; TS, tricuspid valve stenosis; VSD, ventricular septal defect. *Van Praagh classification (1977). Mortality.

TABLE E3. Respiratory complications in heterotaxy patients with CD

	Surgical encounter	pleural effusion	atelectasis	pneumothorax	pneumonedema	pleural exudation	pneumonia	delayed sternal closure
5020	147	1						
5026	370	1				1		1
5027	658							
5028	580					1		
5031	276						1	
	1250	1				1		1
5032	4463	1						
5033	1884							
5038	1642							
	1913		1					
5041	254	1						
	596							
	1891	1		1		1		1
5042	3330				1			1
5043	418							
	1559	1					1	1

5044	658	1						
5048	348							
5053	81							
	2894					1		
5063	148	1				1	1	
5066	1393		1					
	1683	1				1		
5071	56							
5087	937	1				1		
5126	286	1	1				1	
5127	198	1					1	
5129	1324							
	2045	1						
5130	414	1				1		
	1321							
5138	142					1		
5145	1369							
	1703							
	2086							
5204	90					1	1	1
	197						1	
	1243					1		
5635	331					1		
	1012	1				1		1

5707	6			1		1		
	188					1		
5831	125							
	347		1					
	1441	1				1		
51314	469							
51454	267							
	478		1			1		
51485	314					1		
51513	120							
	1238	1				1		
51518	65	1				1		
	375					1		
51531	1647					1		
51569	101					1		
51834	780	1			1	1	1	

Note: 1 refers to respiratory complications occurrence.

TABLE E4. Respiratory complications in heterotaxy patients with Non-CD

	Surgical encounter	pleural effusion	atelectasis	pneumothorax	pneumonedema	pleural exudation	pneumonia	delayed sternal closure
5030	4695							
5037	602							
5046	2376<br>2742							
5049	196		1					
5056	286<br>3226	 1				 1		
5072	253							
5075	1829<br>2185	 1	 1					
5078	203<br>326<br>1413							
5088	728<br>1203<br>2000	 <br>1	 <br>1					
5101	40<br>547					1		1
5102	1437							
5103	211<br>1315					1<br>1		

5115	3017<br>3268					1<br>1		 1
5119	495<br>1777	 1				1<br>1	 1	
5155	87<br>259							
5163	308<br>689<br>1381							
5188	102<br>246					 1		
5191	1129<br>1484							
5238	82							
5309	2640<br>3673						1	
5876	189					1		
5945	762<br>1121					1<br>1		
51039	205		1			1		1
51211	781							
51241	252							
51365	507<br>1020					 1		
51372	53	1			1	1	1	

51468	553							
51486	181<br>1418							 1
51557	303					1		
51766	1653							
51791	1962	1						
51793	554<br>1339	 1				 1	1	

Note: 1 refers to respiratory complications occurrence.
